# Immune response after SARS-CoV-2 vaccination in patients with inflammatory immune-mediated diseases receiving immunosuppressive treatment

**DOI:** 10.1186/s13223-023-00832-0

**Published:** 2023-08-19

**Authors:** Chamaida Plasencia-Rodríguez, Ana Martínez-Feito, Marta Hernández, Lucia Del Pino-Molina, Marta Novella-Navarro, Yolanda Serrano, Miguel González-Muñoz, Diana Peiteado, Gema Bonilla, Irene Monjo, Laura Nuño, Carolina Tornero, Eduardo López-Granados, Alejandro Balsa, Pilar Nozal

**Affiliations:** 1 Rheumatology Unit, La Paz University Hospital, Paseo de la Castellana 261, 28046 Madrid, Spain; 2 Immunology, La Paz University Hospital, Madrid, Spain; 3 Canarias University Hospital, Tenerife, Spain; 4 Center for Biomedical Network Research on Rare Diseases, ISCIII (CIBERER U767), Madrid, Spain; 5 Lymphocyte Pathophysiology in Immunodeficiencies Group, La Paz Institute for Health Research (IdiPaz), Madrid, Spain; 6 Center for Biomedical Network Research on Rare Diseases, ISCIII (CIBERER U754), Madrid, Spain

**Keywords:** Rheumatoid arthritis, SARS-Cov2, Vaccine, Immunosuppressants, Spondyloarthritis, Biologics, TNF inhibitor, Rituximab

## Abstract

**Background:**

Real world data on the response to the SARS-CoV-2 vaccine in patients with immunomediated diseases (IMIDs) treated with immunesuppressants is of great interest because vaccine response may be impaired. The main aim was to study the humoral and cellular immune response after SARS-CoV-2 vaccination in patients with IMIDs treated with immunosuppressants. The secondary aim was to describe the frequency of SARS-CoV-2 infections after vaccination in these patients.

**Material and methods:**

This is an observational study including 86 patients with IMIDs. All patients were treated with biologic or targeted synthetic disease-modifying antirheumatic drugs [b/tsDMARDs: TNF inhibitors (TNFi), rituximab, anti-interleukin 6 receptor (anti-IL6R) or JAK inhibitors (JAKi)]. Demographic and clinical information were collected. After 4–6 weeks of 2nd and 3rd vaccine doses, humoral response was assessed using the Thermo Scientific ELiA SARS-CoV-2-Sp1 IgG Test. Also, in patients with serum SARS-CoV-2 antibody levels under 100UI/ml, cellular response was analyzed using the QuantiFERON SARS-CoV-2 Starter Pack.

**Results:**

A total of 86 patients under b/tsDMARDs and 38 healthy controls were included. Most patients received TNFi (45 with TNFi, 31 with rituximab, 5 with anti-IL6R and 5 with JAKi). SARS-CoV-2 antibodies (Ab) were present in an 86% of patients with IMIDs and in 100% healthy controls (p = 0.017). However, 12 (14%) patients had undetectable SARS-CoV-2 Ab levels, all treated with rituximab. In addition, SARS-CoV-2 Ab (IU/ml) were statistically lower in patients (Mdn (IQR): 59.5 (17–163) in patients vs 625 (405–932) in controls, p < 0.001). Patients treated with rituximab had lower Ab levels than those treated with TNFi and controls (p < 0.001). The cellular response to SARS-CoV-2 vaccine was evaluated in 30 patients. Eleven patients had a positive cellular response, being more frequent in patients treated with rituximab (p = 0.03). SARS-CoV-2 infection was reported in 43% of patients and 34% of controls after vaccination. Only 6 (7%) patients required hospitalization, most of whom treated with rituximab (67%).

**Conclusion:**

SARS-CoV-2 antibody levels were lower in patients than in controls, especially in patients treated with rituximab. A cellular response can be detected despite having a poor humoral response. Severe infections in vaccinated patients with IMIDs are rare, and are observed mainly in patients treated with rituximab.

**Supplementary Information:**

The online version contains supplementary material available at 10.1186/s13223-023-00832-0.

## Introduction

The SARS-CoV-2 pandemic resulted in high morbidity and mortality due to COVID-19. The risk factors associated with worse COVID-19 outcomes among the general population include older age, sex, and chronic diseases [1–3]. Evidence for the severity of SARS-CoV-2 infections in patients with immune-mediated diseases (IMIDs) is controversial [1, 4–6]. Unadjusted analyses from a recent meta-analysis demonstrated that SARS-CoV-2 infection and death are more frequent in patients with IMIDs [1]. EULAR recommendations clarify that patients with IMIDs do not face a higher risk of SARS-CoV-2 infection than individuals without IMIDs and that prognosis is no poorer when they contract it [7]. Although they do emphasize the importance of vaccination prior to starting immunosuppressants or schedule it in patients treated with anti-CD20, in a way to optimize vaccine immunogenicity [7].

Vaccination strategies protect against infections through stimulation of the humoral and cellular immune responses. The relative importance of humoral and cellular immunity in conferring protection from infection varies with the individual microorganism [8]. Antibody responses (seroconversion) are mediated by a B-cell response and are better represented in the literature than T cell-driven responses. The immunogenicity of the SARS-CoV-2 vaccine can be measured based on specific-antibodies to spike protein or T-cell reactivity via the interferon (IFN)-γ response to SARS-CoV-2 peptides [8]. While the role of T-cell responses to SARS-CoV-2 vaccines is not fully understood, emerging evidence suggests that T-cell responses may confer protection [9, 10], even in the absence of a humoral response [11, 12]. However, it is not yet completely known how antibody titers influence the efficacy of these vaccines and how titers decline over time. In addition, the response to a vaccine may be affected by characteristics inherent to the individual, such as age, sex, obesity, and smoking [13].

Vaccination against SARS-CoV-2 has been the largest vaccination program in the history of the Spanish National Health System, and immunocompromised patients have been prioritized in vaccination schedules. The vaccines approved for prevention of SARS-CoV-2 infections include mRNA vaccines (e.g., BNT162b2 and mRNA-1273), non-replicating viral vector vaccines (e.g., Janssen Ad26.COV2.S and AZD1222), and traditional inactivated whole virus vaccines (e.g., CoronaVac). All currently available vaccines against SARS-CoV-2 have proven to be effective in clinical trials. However, their efficacy has not been tested in immunocompromised patients [14]. A recent meta-analysis showed that seroconversion rates and antibody titers after SARS-CoV-2 vaccination are significantly lower in immunocompromised patients than in immunocompetent individuals [14]. Notwithstanding, there is no global consensus on how vaccination strategies should be both in immunocompromised and immunocompetent patients.

Immunosuppressive therapy plays a leading role in the impaired response in patients with IMIDs. Conventional, biologic, and targeted synthetic disease-modifying antirheumatic drugs (c/b/ts-DMARDs) and corticosteroids are widely used in monotherapy or in combination to treat affected patients. Some of these drugs have been shown to impair the response to vaccines [15]. Data on this topic focus largely on influenza, pneumococcal, and tetanus vaccines [8]. In addition, most studies have evaluated the humoral immune response to the above-mentioned vaccines. Whether these data can be extrapolated to provide guidance for vaccination strategies in COVID-19 remains uncertain. Recent data suggest that rituximab, corticosteroids, methotrexate, abatacept, mycophenolate mofetil, and JAK-inhibitors (JAKi) [17] impair SARS-CoV-2 vaccine responses in many patients [8, 16]. The focus has mainly been on patients treated with corticosteroids and anti-CD20 therapy such as rituximab, since this agent has been associated with an increased risk of serious infections and mortality [3, 17, 18].

Although there is increasing evidence on how the response to the SARS-CoV-2 vaccine may be impaired in patients with rheumatic diseases, many areas remain to be clarified. There is an urgent need for real-world data on the immune response to the SARS-CoV-2 vaccine in patients with IMIDs treated with immunosuppressants and on the incidence and severity of SARS-CoV-2 infection in adequately vaccinated patients.

## Methods

### Aim

Our main aim was to study antibody-mediated and cellular-mediated responses after SARS-CoV-2 vaccination in patients with IMIDs treated with different types of immunosuppressive drugs. Our secondary objectives were as follows: to describe the frequency and severity of SARS-CoV-2 infections after vaccination in patients with IMIDs, and to assess the B-cell compartment response after SARS-CoV-2 vaccination in patients treated with rituximab.

### Study design and patients

We performed an ambispective observational study of patients with various IMIDs, namely, rheumatoid arthritis (RA), spondyloarthritis (SpA), psoriatic arthritis (PsA), and connective tissue disease (CTD). Patients were receiving b/tsDMARDs, as follows: TNF inhibitors (TNFi), rituximab, anti-interleukin 6 receptor (anti-IL6R) agents, and JAKi.

Patients were recruited consecutively over 3 months, during which time they were invited to participate at their visits to the clinic (the month before and after the second and third doses of vaccine). The inclusion criteria were as follows: (i) IMIDs treated with b/tsDMARDs; and (ii) availability of laboratory tests 4–6 weeks after the second and/or third dose of the SARS-CoV-2 vaccine. The exclusion criteria were as follows: (i) IMIDs and not having received the second and/or third dose of the vaccine; and (ii) having received the vaccine more than 6 weeks previously.

Demographic, clinical, and treatment data were collected from the electronic clinical records and the database used in the Complex Therapy Unit of the Rheumatology Department. The incidence and severity of SARS-CoV-2 infection after the 2nd and 3rd dose and data on disease flares were also recorded. These data on the incidence of infections and flares were obtained in the successive clinic visits until 6 months after the 3rd vaccine. In case of failing to attend it in person, they were contacted by telephone.

Serum samples were obtained 4–6 weeks after the second or third dose to evaluate the humoral, cell-mediated, and functional response to the vaccine.

### Methods

#### Humoral immune response

The humoral response was assessed in all of the patients included: samples were available for 63 patients after the second and third doses, for 3 patients only after the second dose, and for 20 patients only after the third dose. In addition, 38 controls were selected to compare the humoral response with that of the patients after the second dose.

The humoral response was assessed using fluoroenzyme immunoassay (EliA SARS-CoV-2-Sp1 IgG Test, Thermo Fisher Scientific) with serum samples. The lower limit of detection was 0.7 IU/ml, and the positive cut-off was set at 10 IU/ml. The upper limit of detection was 204 IU/ml. Data from IMIDs patients were compared with those from the 38 healthy controls analyzed 4 weeks after the second vaccine dose. In this cohort, samples above 204 IU/ml were subsequently diluted to obtain a final antibody concentration.

#### Cell-mediated immune response

The cellular response was evaluated only in patients with a poor humoral response, which was defined as IgG antibody levels against SARS-Cov2 < 100 IU/ml, using a QuantiFERON SARS-CoV-2 (QTF-SARS-COV-2) Starter Pack (Qiagen). A cut-off value of 0.15 IU/ml was applied to differentiate between positive and negative cell-mediated immune responses.

#### Characterization of the B-lymphocyte compartment in patients with RA treated with rituximab

Additionally, we investigated in detail the pre-germinal center (GC) B-cell compartment using flow cytometry. Samples were available in 13 (43%) of the 31 rituximab-treated patients but only were selected 7 RA patients who had more than 1% of total CD19^+^ B cells in peripheral blood. The B-cell phenotype in RA patients was compared with our 62 previously characterized HC cohort [19]. To that end, we used the pre-GC B-cell tube according to the EuroFlow standard operating procedures for sample preparation and data acquisition and analysis developed by EuroFlow [20, 21]. The pre-GC B-cell tube makes it possible to identify immature/transitional B cells, mature naïve B lymphocytes, unswitched memory B cells (MD^+^) (CD27^+^ CD38^lo^ CD5^−^ CD24^het^ smIgM^++^D^+^), and switched memory B cells (MD−) (CD27^+/^− CD38^lo^ CD5− CD24^het^ smIgM−D−). Immature/transitional B cells are sub-classified according to the expression of CD5, CD38, CD21, and CD24 into 3 subsets of increasingly mature B lymphocytes: CD− CD38^++^ CD21^het^ CD24^++^; CD5^+^ CD38^+/++^ CD21^het^ CD24^++^; and CD5^+^ CD38^het^ CD21^+^ CD24^+^ (immature/transitional B lymphocytes). Mature naïve B lymphocytes are further divided into 3 subsets according to CD21 and CD24 expression into the following mature naïve B cells: CD21^+^ CD24^+^; CD21− CD24^++^; and CD21− CD24− (see Additional file 1: Fig. S1).

Total absolute counts of B-cell subsets in RA patients were compared using normal reference ranges of absolute counts in healthy controls, as previously described [19].

### Statistical analysis

First, descriptive analyses were performed for the demographic and clinical variables. The results are shown as mean and standard deviation (SD) or median and interquartile range (IQR) depending on the normality of the distribution for continuous variables and as relative frequencies for categorical variables. The frequency data were compared using the Pearson chi-squared or Fisher exact test. Unpaired continuous data were compared using the unpaired *t* test or Mann–Whitney test, depending on the data distribution. For multiple comparisons, one-way ANOVA or the Kruskal–Wallis test was used, again, depending on the data distribution. Additionally, multiple comparisons were adjusted using the Bonferroni test.

Second, associations between the humoral immune response to SARS-COV-2 vaccine, clinical variables, and treatments were evaluated using univariate and multivariate logistic regression models, and data were presented as the odds ratio (OR) and 95% confidence interval (CI). Variables with a p-value < 0.1 in the univariate analysis were selected for the multivariate analysis. The presence of collinearity between covariates was tested. In case of no interaction, the model was subsequently adjusted for these covariates.

## Results

### Patient characteristics

Of the 885 patients with IMIDs receiving active treatment with b/tsDMARDs in the Complex Therapy Unit until April 2022, 86 (50 RA, 20 SpA, 10 PsA, and 6 CTD) were included in this study, along with 38 healthy controls. The characteristics of the patients and controls are shown in Table 1. Most patients (45/86) received TNFi, 31 were treated with rituximab, 5 with anti-IL6R agents, and 5 with JAKi.

**Table 1:** Patient characteristics

	Controls n = 38	All patients N = 86	TNFi n = 45	RTX n = 31	Anti-IL6R n = 5	JAKi n = 5
Demographic and clinical characteristics						
Sex (female)	30 (79%)	55 (64%)	19 (42%)	27 (87%)	4 (80%)	5 (100%)
Age	48 ± 14	56 ± 14*	53 ± 13	61 ± 12	52 ± 22	56 ± 10
BMI	24 ± 2.4	27 ± 6.1*	26 ± 6.4	27 ± 5.4	26 ± 8.5	21 ± 1.6
Comorbidities						
Diabetes	0 (0%)	1 (1.2%)	1 (2.2%)	0 (0%)	0 (0%)	0 (0%)
Arterial hypertension	7 (18%)	29 (34%)	12 (27%)	15 (48%)	1 (20%)	1 (20%)
Current smokers	4 (12%)	12 (14%)	5 (31%)	5 (31%)	1 (6.3%)	1 (6.3%)
Chronic lung disease	4 (12.5%)	2 (2.3%)	0 (0%)	2 (6.5%)	0 (0%)	0 (0%)
Dyslipidemia	11 (29%)	26 (30%)	13 (29%)	13 (42%)	0 (0%)	0 (0%)
Diagnosis						
RA	–	50 (58%)	15 (33%)	27 (87%)	3 (60%)	5 (100%)
SpA	–	20 (23%)	20 (45%)	0 (0%)	0 (0%)	0 (0%)
PsA	–	10 (12%)	10 (22%)	0 (0%)	0 (0%)	0 (0%)
CTD	–	6 (7%)	0 (0%)	4 (13%)	2 (40%)	0 (0%)
Serology findings						
RF+	–	45 (47%)	13 (29%)	25 (83%)	2 (40%)	5 (100%)
ACPA+	–	47 (55%)	14 (34%)	25 (86%)	3 (75%)	5 (100%)
HLA-B27+	–	14 (16%)	14 (47%)	0 (0%)	0 (0%)	0 (0%)
ANA+	–	19 (22%)	5 (12%)	11 (38%)	3 (40%)	0 (0%)
Treatment						
Methotrexate use	–	63 (73%)	23 (51%)	16 (52%)	2 (40%)	4 (80%)
Methotrexate dose (mg/week)	–	16.5 ± 6.1	16 ± 6.5	17.5 ± 5.5	17.5 ± 3.5	13 ± 8
Prednisone use	–	23 (27%)	6 (13%)	14 (45%)	2 (40%)	1 (20%)
Prednisone dose (mg/day)	–	3.14 ± 2.1	1 ± 1.1	3.6 ± 1.6	6.3 ± 1.7	2.5 ± 3.5
Time under b/tsDMARD	–	12.1 ± 14.7	10.3 ± 7.4	17 ± 9.2	17 ± 13.2	16 ± 3.8

Data for sex, comorbidities, diagnosis, serological findings, and use of methotrexate andcorticosteroids are expressed as n (%). Data on age, BMI, and methotrexate and corticosteroid doses are expressed as mean ± SD

*The comparison between controls and patients revealed statistically significant differences (p < 0.05)

Overall, patients were older and had a higher BMI than controls (see Table 1). A sub-analysis was performed to assess whether these differences could be attributed to specific diagnoses (see Additional file 1: Table S1). Differences in age were mainly attributed to the RA group. Patients with PsA and RA had the highest BMI.

All healthy controls and most patients (91%) received 3 doses (see Additional file 1: Table S2). Vaccination patterns are specified in detail according to treatment group in Additional file 1: Table S2. The most common pattern in patients was all 3 doses with Pfizer, followed by Astra Zeneca + Pfizer + Moderna.

### Humoral immune response to the SARS-Cov2 vaccination

**Fig. 1:**
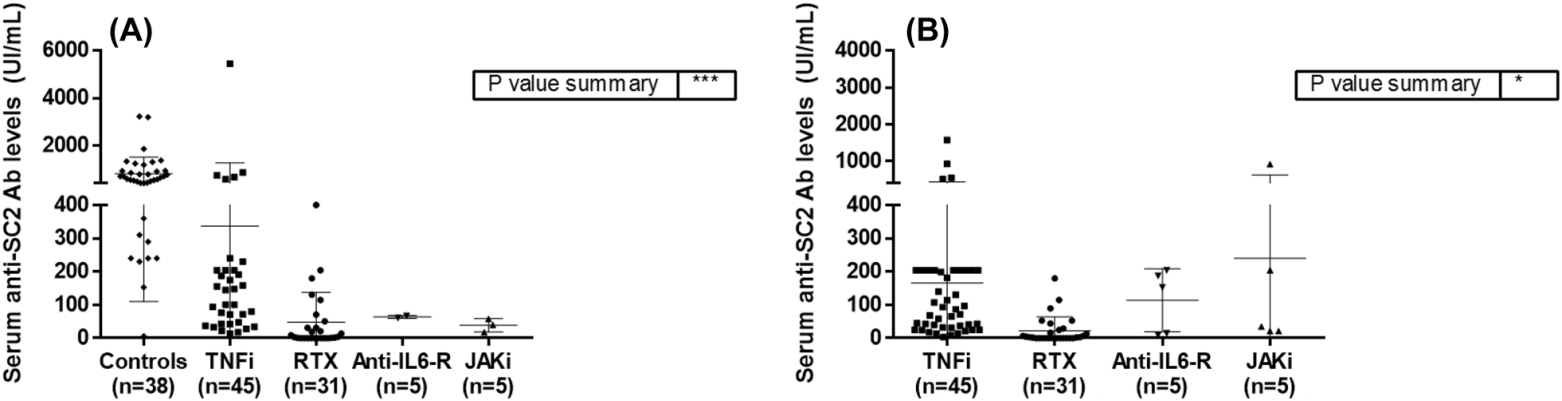
Humoral immune response after the second and third vaccine doses. **A** Comparison of anti-SARS-CoV-2 Ab levels between controls and treatment groups after the second vaccine dose. **B** Comparison of anti-SARS-CoV-2 Ab levels between treatment groups after the third vaccine dose

After vaccination, anti-spike IgG antibodies against SARS-COV-2 were detected in 74 of the 86 patients with IMIDs (86%) and in all 38 of the healthy controls (100%) (p = 0.017). In addition, serum anti-SARS-COV-2 antibody levels were statistically significantly lower in patients than in healthy controls regardless of the diagnosis (median [IQR]: 47 [8–146] IU/ml in RA, 129 [44–197] IU/ml in SpA, 87 [24–602] IU/ml in PsA, 3 [0.4–47] IU/ml in CTD, 625 [405–932] IU/ml in controls; p < 0.0001) (Additional file 1: Fig. S2). Table 2 and Fig. 1 show serum anti-SARS-COV-2 antibody levels in each therapy group and separately by vaccine dose. Regardless the vaccination dose, the lowest anti-SARS-COV-2 antibody levels were recorded in patients who received rituximab (p < 0.00005). Indeed, 70% of patients had low antibody levels (< 100 IU/mL) after the 2nd or 3rd dose of the vaccine. Additional file 1: Table S3 shows the percentage of patients with low anti-SARS-COV-2 antibody levels in each therapy group.

**Table 2:** Humoral immune response after the second and third doses of SARS-CoV-2 vaccine

Serum anti-SARS-Cov-2 antibody levels (IU/ml) after the 2nd vaccine						
Controls n = 38	All patients n = 66	TNFi n = 34	RTX n = 27	Anti-IL6R n = 2	JAKi n = 3	*p*
625 (405–932)	94 (42–204)	123 (43–204)	4 (0–51)	64 (61–64)	40 (18–58)	< 0.0005

Serum anti- SARS-CoV-2 antibody levels (IU/ml) after the 3rd vaccine						
	All patients N = 83	TNFi n = 44	RTX n = 29	Anti-IL6R n = 5	JAKi n = 5	
–	40 (6.20–153)	80 (31–204)	3 (0–27)	153 (12–196)	35 (21–562)	0.03

Anti-SARS-CoV-2 antibody levels were compared between controls and patients only after the second dose of the vaccine. Of the total of 86 patients, 66 and 83 patients had samples available after the second and third doses of vaccine, respectively. Patients treated with anti-IL6R agents and JAKi were not included in the statistical comparisons owing to the small sample size. All data are expressed as median (IQR)

Serum anti-SARS-COV-2 antibody levels were undetectable in 12 patients (14%) after the third dose and in 9 after the second dose. All these patients were treated with rituximab (10 RA and 2 CTD). None of the 9 patients seroconverted between the second and third doses. However, among the 54 patients who seroconverted after the 2nd dose and had samples available after the 3rd dose, it was observed that the antibody levels were significantly lower after the 3rd dose (median [IQR]: 204 [47–530] IU/ml after 2nd dose vs 43 [16–140] IU/ml after 3rd dose, p = 0.041).

A regression analysis was performed to assess which factors were associated with undetectable levels of anti-SARS-COV-2 antibody. In the multivariate analysis, only receiving rituximab was associated with lack of humoral response (OR: 24.7; see Table 3).

**Table 3:** Factors associated with the absence of humoral immune response to the SARS-CoV-2 vaccination

	Univariate analysis		Multivariate analysis	
	OR	95% CI	OR	95% CI
Age	0.9	0.9–1.01	0.9	0.9–1.04
BMI	0.9	0.9–1.1	–	–
Rituximab use	34*	4.1–280.2*	24.7*	2.9–211.2*
Methotrexate use	1.3	0.4–4.3	–	–
Methotrexate dose (mg/week)	0.9	0.8–1.1	–	–
Prednisone use	4.2*	1.2–14*	2.2	0.5–9.3
Prednisone dose (mg/day)	0.9	0.6–1.3	0.3	0.1–1.9

The univariate analysis revealed that sex, age, RA diagnosis, rituximab and prednisone use were associated with undetectable serum levels of anti-SARS-CoV-2 antibodies. However, the multivariate analysis only showed an association with rituximab use

^*^ Statistically signicant data are shown in bold.

### Cell-mediated immune response to the SARS-COV-2 vaccination

The cell-mediated immune response was studied in 30 patients with a poor humoral immune response (anti-SARS-COV-2 antibody levels < 100 IU/ml) after the second dose. Most were treated with rituximab (18 with rituximab, 10 with TNFi, and 2 with JAKi).

**Fig. 2:**
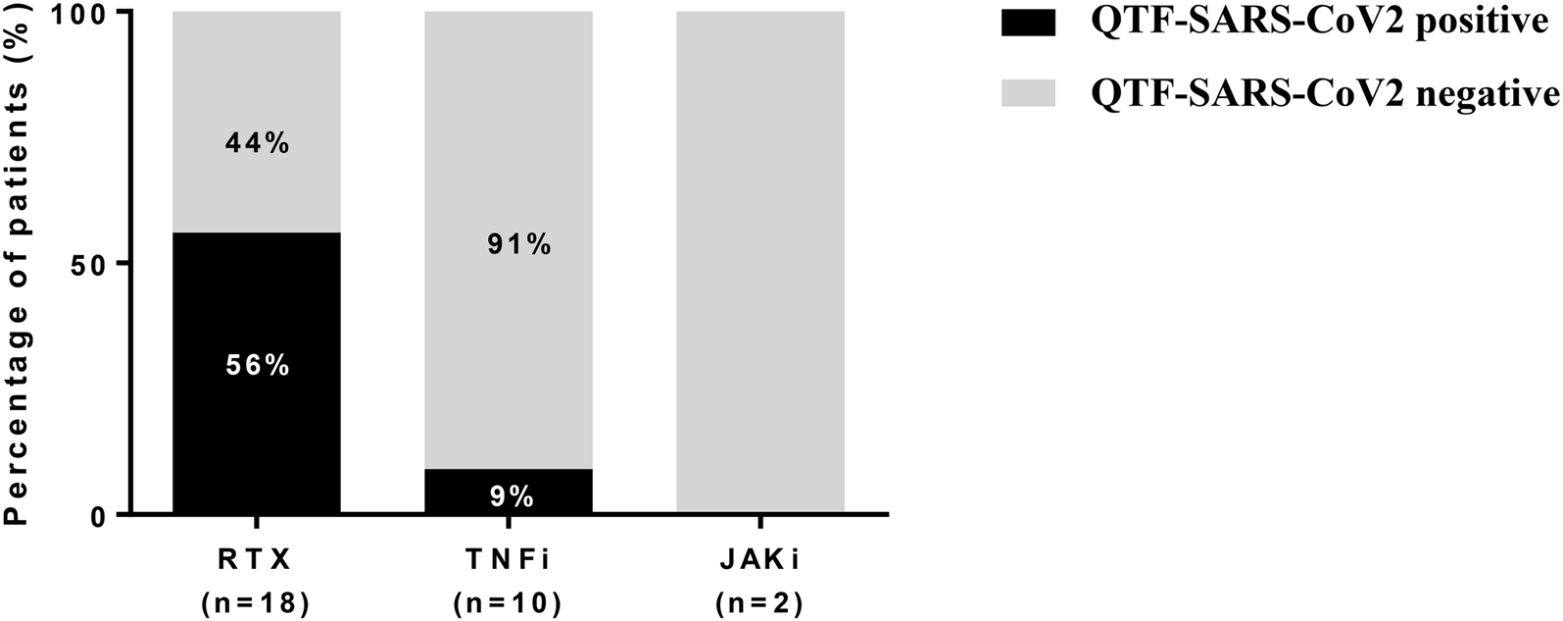
Cellular immune response after the second and third vaccine dose. Percentage of patients with a positive or negative cell-mediated immune response after two doses according to treatment. *RTX* rituximab, *TNFi* TNF inhibitors, *JAKi* JAK inhibitors

Cell-mediated responses to SARS-COV-2 were positive in 11 patients: 10 of 18 patients receiving rituximab (56%), 1 of the 10 patients treated with TNFi (9%), and in none of those receiving JAKi (Fig. 2). Thus, the differences in cell-mediated response were statistically significant between the rituximab and TNFi groups (p = 0.03).

### Characterization of the B-lymphocyte compartment in RA patients treated with rituximab prior to SARS-COV-2 vaccination

Of the 31 patients treated with rituximab, samples for cytometry were collected in 13 patients (42%) with RA. Of these, only 7 patients with RA had more than 1% of total CD19+ B cells in peripheral blood were detected. If disease activity was well controlled, the rituximab infusion was delayed more than 6 months to first schedule the first and second vaccinations. The median time between the last dose of rituximab and the first dose of SARS-COV-2 vaccine was 1.15 (1.04–2.12) year.

Flow cytometry revealed CD19^+^ B cells in 7 patients (54%), all of whom had detectable anti-SARS-COV-2 antibodies. The cellular immune response was analyzed in 4 patients, with QTF-SARS-COV-2 being positive in only 1 of them.

**Fig. 3:**
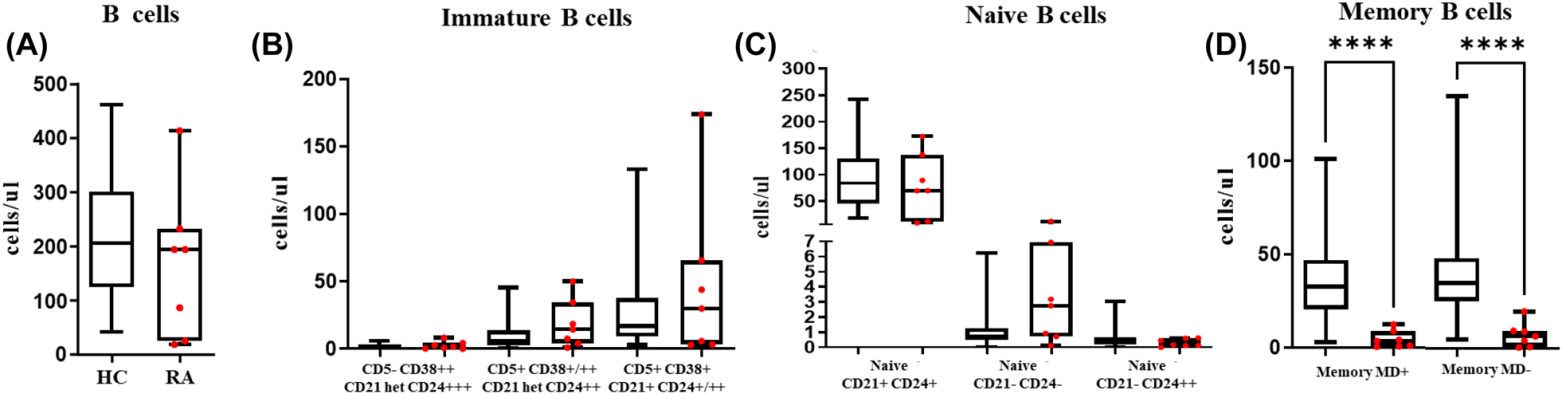
Characterization of B cells in patients with RA treated with rituximab. Absolute counts of major pre-GC and post-GC B-cell subsets are shown using box and whiskers plots separately for HC (n = 62) in black and RA patients (n = 7) red dots, where horizontal lines and vertical lines represent the median and both 5th and 95th percentile values, respectively

Some RA patients had normal absolute total CD19^+^ B cell counts, whereas others presented considerably lower counts than healthy controls (Fig. 3A). In addition, a relevant observation in the group of patients is that, the CD19^+^ B cell counts levels were higher in those with a longer time since the last rituximab infusion (Mdn (IQR): 42 (20–81) cells/µl in patients with last infusion < 1.15 years versus 194 (117–231) cells/µl in patients with last infusion ≥ 1.15 years, p = 0.034). Additionally, the humoral response was also higher in patients with delayed RTX infusion [after 2nd dose: (Mdn (IQR): 1.35 (0–67) IU/ml in patients with last infusion < 1.15 years versus 26 (2.7–104.25) IU/ml in patients with last infusion ≥ 1.15 years, p = 0.037); after 3^rd^ dose: (Mdn (IQR): 0.95 (0–13.75) IU/ml in patients with last infusion < 1.15 years versus 27 (11.27–74.75) IU/ml in patients with last infusion ≥ 1.15 years, p = 0.039)].

Further characterization of the pre-GC B-cell compartment, which includes the immature/transitional and naïve B-cell subsets, had shown a slight expansion of the less differentiated CD5− CD38^++^ CD21^het^ CD24^++^ immature/transitional B lymphocytes RA patients (1.76 vs 0.89 cells/µL in healthy controls) and in the other 2 more differentiated immature/transitional B-cell subsets: CD5^+^ CD38^+/++^ CD21^het^ CD24^++^ (14.59 vs 5.60 cells/µl in HD) and CD5^+^ CD38^het^ CD21^+^ CD24^+^ (30.14 vs 16.90 cells/µl in HD) (Fig. 3B). Regarding the naïve B-cell compartment, RA patients were characterized by a slight expansion of CD21−CD24− (2.75 vs 0.74 cells/µl) and CD21−CD24^++^ naïve B cells (0.40 vs 0.17 cells/µl) (Fig. 3C). Finally, regarding post-GC memory B cells, RA patients displayed a significant reduction (vs. healthy controls) in unswitched IgMD+ memory B cells (3.38 vs 32 cells/µl, p < 0.0001) and switched IgMD+ memory B cells (6.56 vs 35 cells/µl, p < 0.0001) (Fig. 3D).

### Clinical events after SARS-COV-2 vaccination

No differences were observed in the frequency of SARS-COV-2 infection between patients and healthy controls after the second and third doses (43% patients vs 34% HC, p = 0.356). The frequency of SARS-COV-2 infection was similar in each treatment group: 58% (19/45) for TNFi, 55% (14/31) for rituximab, 60% (2/5) for anti-IL6R, and 60% (2/5) for JAKi (see Additional file 1: Fig. S1). Most patients had 1 SARS-COV-2-infection (92%) and only 3 patients had 2 SARS-COV-2 infections. No healthy controls required hospitalization. Six patients (7%) required hospitalization between the second and third doses, and most of them were receiving rituximab (67%). All admitted patients had pneumonia. Only 1 patient was admitted to the intensive care unit after the second dose. This patient received rituximab, and a humoral immune response was not detected.

Disease flares after SARS-COV-2 infection were detected in 3 patients (1 with TNFi and 2 with rituximab). Disease activity was controlled by adjusting treatment with corticosteroids, although 1 patient required a change in biological treatment.

## Discussion

This study provides insight into the clinical relevance of immune response to SARS-COV-2 vaccination in patients with IMIDs treated with immunosupressants. In our cohort, most of patients (86%) with IMIDs treated with b/tsDMARDs had detectable humoral responses. However, anti-SARS-COV-2 Ab levels were lower in patients than in controls, with rituximab being associated with a higher risk of an absent humoral response (OR: 18.5), as expected. On the other hand, we found that up to 37% of patients with a poor humoral response may have a cellular response to the vaccine, being more frequently detected in patients treated with rituximab (91%). Another important issue is that the presence of CD19+ B cells in peripheral blood is associated with the time since the last rituximab infusion. The study of the pre-GC B cell compartment showed that the B cell regeneration pattern in patients is mainly at the expense of immature cells.

There is substantial variation in the immune response to vaccination between individuals. Factors that influence humoral and cellular vaccine responses include intrinsic host factors such as age, sex, genetics, and comorbidities, perinatal factors such as gestational age, birth weight, feeding method, and maternal factors, as well as extrinsic factors like preexisting immunity, microbiota, infections, and antibiotics [13]. Additionally, environmental factors including geographic location, season, family size, and toxins, behavioral factors such as smoking, alcohol consumption, exercise, and sleep, and nutritional factors such as body mass index, micronutrients, and enteropathy can also influence how individuals respond to vaccines [13]. Sex differences in the response to vaccination have been reported [22, 23]. Females generate overall higher antibody levels, experience more adverse events, have higher B cell frequencies and exhibit elevated innate immune cell phagocytic activity [22]. In contrast, males have increased NK cell numbers, enhanced type-1 immune responses, and so on [22]. However, in our study we have observed that women are associated with lower levels of antibodies. These discrepancies are due to the fact that most of the patients are female and the lowest levels were found in patients with RA treated with rituximab. The impact of the BMI on the response to vaccines is more controversial [24–26]. Bates et al. found no association between antibody titers and BMI at 50 and 200 days after vaccination against SARS-COV-2 in 127 people [24]. In our cohort, RA patients had a higher BMI but this was not independently associated with lack of humoral response.

Moreover, factors related with vaccine (such as vaccine type, product, adjuvant, and dose) and administration (schedule, site, route, time of vaccination, and coadministered vaccines and other drugs) are also important [13, 27]. An understanding of all these factors and their impacts on the design of vaccine studies and decisions on vaccination schedules can help to improve vaccine immunogenicity and efficacy. In fact, in a study carried out in the Swiss cohort (SCQM) including 565 patients, two-dose vaccination with mRNA1273 (Moderna) versus BNT162b2 (Pfizer) resulted in a higher anti-SARS-CoV-2 Ab levels [27]. In our study most patients (50) received the vaccination scheme with BNT162b2 (Pfizer) and only 8 patients were vaccinated with mRNA1273 (Moderna) as the first 2 doses. Thus, statistical significance analyses regarding vaccine type could not be performed in our study.

The seroconversion rate in our cohort of patients with IMIDs was very high (86%). However, anti-SARS-CoV-2 Ab levels were lower in patients with IMIDs treated with b/tsDMARDs than in controls. These data are consistent with previous publications [8]. In an observational multicenter study, researchers observed that BNT162b2 (Pfizer) was immunogenic in most patients with IMIDs with a good safety profile. In parallel, they found that drugs such as rituximab, mycophenolate, glucocorticoids, and abatacept were associated with a lower humoral response to the vaccine [8, 15]. These findings coincide with our data, which show that the lowest levels of antibodies were detected in patients with rituximab. Furthermore, all patients with undetectable levels of anti-SARS-CoV-2 Ab in our cohort were treated with this drug. A recent review found that patients with SLE have lower rate of seroconversion than healthy controls and patients with RA [28]. In the present study, we only included 6 patients with connective tissue disorders, so statistical analysis comparing with other pathologies are not able to be performed. However, it is worth emphasizing that anti-SARS-CoV-2 Ab levels were lower in patients with CTD than in the other pathologies (see Additional file 1: Fig. S2).

Another interesting point to describe in detail is the cellular response that patients with IMIDs can generate against the SARS-COV-2 vaccine. Lledó et al. found that cellular immune response against SARS-CoV-2 was similar between patients with IMIDs (53) and controls (61) [29]. They also observed that this response was not affected by the different pathologies or by immunosuppressive treatments. Another recent study by Bock et al. evaluated the humoral and cellular response to the SARS-CoV-2 vaccine in patients with multiple sclerosis treated with immunosuppressant [30]. First, they found that patients treated with anti-CD20 therapy had a poor humoral response but an intact cellular response. In contrast, an impaired cellular and humoral response was seen in patients treated with sphingosine 1 phosphate inhibitors [30]. This may be due to the direct action of this drug on T lymphocytes, secondarily affecting the T-cell dependent B-cell activation. Our results are in agreement with findings observed in this last study. We found that the cellular response was more frequent in patients with anti-CD20 therapy than in those with anti-TNF therapy and with JAK inhibitors. These differences could be due to the type of assay used to evaluate the cellular response. Whereas Lledó et al. used the ELISpot assay to evaluate the cellular response, Bock et al. and our study used the QTF-SARS-COV-2 assay. Both assays evaluated the same immune response but have different nuances. ELISpot is performed with PBMC and measures interferon-producing cells after a stimulus, while the QTF-SARS-COV-2 measures serum interferon production after a stimulus [31]. Another aspect to consider is that Lledó et al. included a small number of patients treated with TNFi and rituximab (10 and 4 patients, respectively). A possible hypothesis that supports our findings in TNFi group lies in the influence of these drugs on the homeostasis of T lymphocytes regulating their response in many ways [32]. Hence, it is logical that in patients treated with TNFi, the cellular response to vaccines may be impaired.

Delving into the mechanism of rituximab, CD20 is expressed on circulating peripheral blood B cells, but not on bone marrow stem cells or plasma cells [33]. Therefore, anti-CD20 drugs would not be expected to deplete the total B-cell pool, and theoretically, should not compromise humoral immunity as B-cell regeneration from bone marrow precursors is not directly inhibited. B-cell recovery upon treatment discontinuation is quite heterogeneous, and mild to profound impact on the peripheral B-cell compartment can be observed, together with partial or severe impairment of the antibody response. Here, in fact we observed RA patients with almost normal absolute counts of total B-cells whereas others presented reduced B-cells or undetectable B-cell counts. In general, B-cell numbers remains reduced or undetectable in peripheral blood up to 2–6 months post-rituximab [34]. B-cell reconstitution may take longer to reach pretreatment levels, depending on underlying clinical context, medication dosing, and treatment duration, concomitant administration of immunosuppressive medications, age and patient comorbidities [35]. Here, RA patients presented increased absolute numbers of the three immature/transitional B-cell subsets that are the first populations of maturation that appear in peripheral blood during reconstitution after rituximab as previously observed [36] (Fig. 3B and Additional file 1: Fig. S3). In addition, the expansion of CD21^−^ B-cells have been described in the naïve B-cell compartment and in the memory B cell (MBC) compartment [37]. Here, the MBC is severely reduced, but within the naïve B-cell compartment we also detected in some patients the expansion of CD21^−^ CD24^−^ mature naïve B cells. Although we have not evaluated the B-cell phenotype prior to the rituximab treatment, it could be presumed that the regeneration of B-cell compartment, with accumulation of CD21^−^ B-cells, is similar to the preexisting one which is the result of the chronic stimulation occurred in the pathogenesis of RA.

In concordance with delayed MBC compartment reconstitution, seven patients with RA presented severely reduced unswitched IgMD^+^ MBC and switched IgMD^+^ MBC, as also previously reported in systemic lupus erythematous (SLE) patients treated with rituximab [38]. Considering all, a message that we take from the basic to the clinic, is that delaying the infusion of rituximab prior to vaccination enables the regeneration of B cell compartment thus favoring a better vaccination response.

A very reassuring fact is that in our cohort there is no difference in the frequency of infections between patients and controls after vaccination and they were mostly mild cases. The infections requiring hospitalization after vaccination were scarce and they only occurred in patients who were treated with rituximab. These results support the importance of promoting an adequate vaccination strategy as stated in the EULAR recommendations for vaccination in patients with IMIDs [7]. However, many unanswered questions remain. In this work we have observed that the majority of patients with IMIDs treated with b/tsDMARD had detectable anti-SARS-COV-2 antibodies and infections in vaccinated patients were mostly mild.

Following the recommendations from the Ministry of Health, all patients received additional COVID-19 vaccine boosters (third, fourth and fifth doses). It is evident that vaccination against SARS-COV-2 has decreased the frequency and severity of infections. In fact, our data show that the majority of patients presented mild infections after vaccination like those observed in the general population. However, an interesting point observed in this work is that the antibody titers were significantly lower after the 3rd dose than after the initial regimen that included the 1st and 2nd doses. This fact reflects how the antibody response diminishes over time and may be related to the dose used and/or the timing of the booster. Therefore, the frequency and number of boosters needed for these patients are unclear. In this sense, efforts should be made to move towards more personalized strategies, establishing profiles of patients at higher risk based on scientific evidence.” In this sense, efforts should be made to move towards more personalized strategies, establishing profiles of patients at higher risk based on scientific evidence.

Some limitations were observed in the present study. First, the limited number of patients in the groups of patients treated with anti-IL6R and JAKi. Second, some differences in age and BMI between controls and patients. However, these age differences were only observed in patients with RA. Regarding BMI, recent data do not seem to find a relationship between BMI and response to the SARS-COV-2 vaccine. Another aspect to consider is that the cellular response was only studied in patients with a poor humoral response, so we cannot compare the cellular response between patients with a good humoral response or not.

## Conclusions

On brief, the humoral immune response to the SARS-COV-2 vaccine is poorer in patients than in healthy controls, especially in those treated with rituximab. However, in patients with rituximab, a cellular-mediated immune response can be detected despite having a poor humoral response. Finally, severe infections in vaccinated patients with IMIDs are rare, mainly observed in patients treated with rituximab.

In conclusion, a scheduled delay of the rituximab infusion to ensure a better humoral response to the vaccine would be recommended in patients with IMID.

### Supplementary Information

**Additional file 1: Table S1.** Comparison of characteristics between controls and patients classified by diagnosis. **Table S2.** SARS-CoV-2 vaccination patterns in patients and controls. **Table S3.** Comparison of patients with low anti-SARS-Cov-2 antibody levels (< 100 IU/ml) in each therapy group. **Figure S1.** Distribution of B-cell subsets by degree of maturation, including immature cells (CD5− CD38++ CD21het CD24++, CD5+ CD38+/++CD21het CD24++ and CD5+ CD38het CD21+ CD24+), naïve cells (CD21+ CD24+, CD21−CD24− and CD21− CD24++), and memory B cells (MBC) in peripheral blood. **Figure S2.** Humoral immune response after the second vaccine dose in patients with IMIDs. Comparison of the second vaccine dose between controls and patients. **Figure S3.** Proportion of patients with SARS-CoV-2 infections in each therapy group. This graph shows the proportion of patients who developed SARS-CoV-2 infection and its severity after the second dose of vaccine according with each treatment.

## Data Availability

Data can be provided upon request to the authors.

## Abbreviations

IMIDs: Immune-mediated diseases
RA: Rheumatoid arthritis
SpA: Spondyloarthritis
PsA: Psoriatic arthritis
CTD: Connective tissue disease
BMI: Body mass index
c/b/ts-DMARDs: Conventional, biologic, and targeted synthetic disease-modifying antirheumatic drugs
JAKi: JAK-inhibitors
TNFi: TNF inhibitors
anti-IL6R: Anti-interleukin 6 receptor
SLE: Systemic lupus erythematous
