# Supplementary material for: Immune response after SARS-CoV-2 vaccination in patients with inflammatory immune-mediated diseases receiving immunosuppressive treatment
Source: Allergy Asthma Clin Immunol. 2023 Aug 19;19:71. doi: 10.1186/s13223-023-00832-0 (PMC10439605; doi:10.1186/s13223-023-00832-0)
Supplement: Supplementary file 1 — Additional file 1: Table S1. Comparison of characteristics between controls and patients classified by diagnosis. Table S2. SARS-CoV-2 vaccination patterns in patients and controls. Table S3. Comparison of patients with low anti-SARS-Cov-2 antibody levels (< 100 IU/ml) in each therapy group. Figure S1. Distribution of B-cell subsets by degree of maturation, including immature cells (CD5− CD38++ CD21het CD24++, CD5+ CD38+/++CD21het CD24++ and CD5+ CD38het CD21+ CD24+), naïve cells (CD21+ CD24+, CD21−CD24− and CD21− CD24++), and memory B cells (MBC) in peripheral blood. Figure S2. Humoral immune response after the second vaccine dose in patients with IMIDs. Comparison of the second vaccine dose between controls and patients. Figure S3. Proportion of patients with SARS-CoV-2 infections in each therapy group. This graph shows the proportion of patients who developed SARS-CoV-2 infection and its severity after the second dose of vaccine according with each treatment. [file 13223_2023_832_MOESM1_ESM.docx]

**Table S1. Comparison of characteristics between controls and patients classified by diagnosis.**

|  | **Controls**  **n=38** | **RA**  **n=50** | **SpA**  **n=20** | **PsA**  **n=10** | **CTD**  **n=6** | **p** |
| --- | --- | --- | --- | --- | --- | --- |
| **Demographic and clinical characteristic** | | | | | | |
| Age | 48±14 | **60±12** | 50±15 | 54±14 | 43±17 | <0.0001 |
| BMI | 24±2.4 | **27±5.6** | 25±3.9 | **30±9.7** | 20±2.7 | <0.0001 |

**Legend:** Data on age and BMI are compared with controls and diagnosis.

**Table S2. SARS-CoV-2 vaccination patterns in patients and controls.**

|  | **Controls**  **n=38** | **All patients**  **n=86** | **TNFi**  **n=45** | **RTX**  **n=31** | **Anti-IL6R**  **n=5** | **JAKi**  **n=5** |
| --- | --- | --- | --- | --- | --- | --- |
| **Patients with 2 doses of vaccine** | | | | | | |
| PF-PF | 0 (0%) | 4 (4.4%) | 1 (2%) | 2 (6%) | 1 (%) | 0 (%) |
| MD-MD | 0 (%) | 2 (2.3%) | 1 (2%) | 1 (3%) | 0 (%) | 0 (%) |
| AZ-AZ | 0 (%) | 2 (2.3%) | 0 (0%) | 2 (6%) | 0 (%) | 0 (%) |
| **Patients with 3 doses of vaccine** | | | | | | |
| PF-PF-PF | 38 (100%) | 46 (53%) | 23 (51%) | 20 (65%) | 2 (40%) | 1 (20%) |
| PF-PF-MD | 0 (%) | 3 (4%) | 2 (4%) | 1 (1%) | 0 (%) | 0 (%) |
| AZ-PF-MD | 0 (%) | 23 (27%) | 14 (31%) | 5 (16%) | 2 (40%) | 2 (40%) |
| MD-MD-PF | 0 (%) | 6 (7%) | 4 (10%) | 0 (0%) | 0 (%) | 2 (40%) |

**Legend:** The type and number of vaccinations administered throughout this study are listed. PF=Pfizer, AZ= Astra Zeneca, MD= Moderna.

**Table S3. Comparison of patients with low anti-SARS-Cov-2 antibody levels (<100 IU/mL) in each therapy group.**

| **Serum anti-SARS-Cov-2antibody levels <100 IU/mL after the 2^nd^ vaccine** | | | | | |
| --- | --- | --- | --- | --- | --- |
| **All patients**  **n=66** | **TNFi**  **n=34** | **RTX**  **n=27** | **Anti-IL6R**  **n=2** | **JAKi**  **n=3** | ***p*** |
| 46 (70%) | 17 (50%) | 23 (85%) | 2 (100%) | 3 (100%) | <0.010 |
| **Serum anti-SC2 antibody levels <100 IU/mL after the 3^rd^ SC2 vaccine** | | | | | |
| **All patients**  **N=83** | **TNFi**  **n=44** | **RTX**  **n=29** | **Anti-IL6R**  **n=5** | **JAKi**  **n=5** |  |
| 57 (69%) | 25 (57%) | 27 (93%) | 2 (40%) | 3 (60%) | 0.005 |

**Figure S1**. **Distribution of B-cell subsets by degree of maturation**, including immature cells (CD5– CD38++ CD21het CD24++, CD5+ CD38+/++ CD21het CD24++ and CD5+ CD38het CD21+ CD24+), naïve cells (CD21+CD24+, CD21-CD24– and CD21– CD24++), and memory B cells (MBC) in peripheral blood.


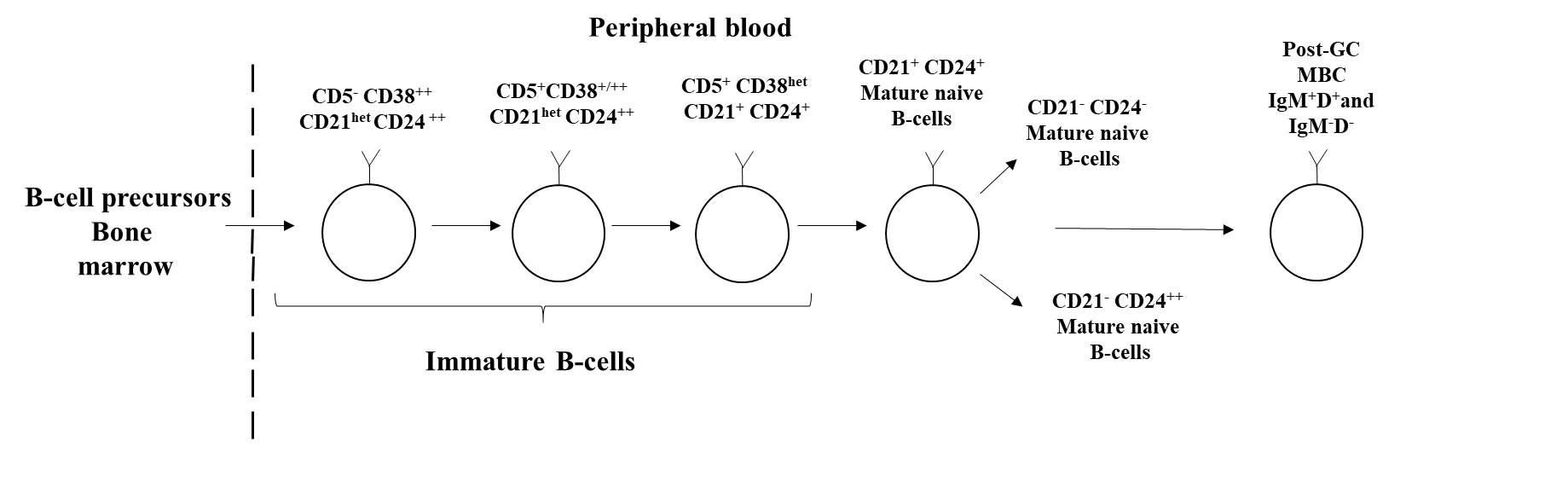


**Figure S2. Humoral immune response after the second vaccine dose in patients with IMIDs.** Comparison of the second vaccine dose between controls and patients.

**
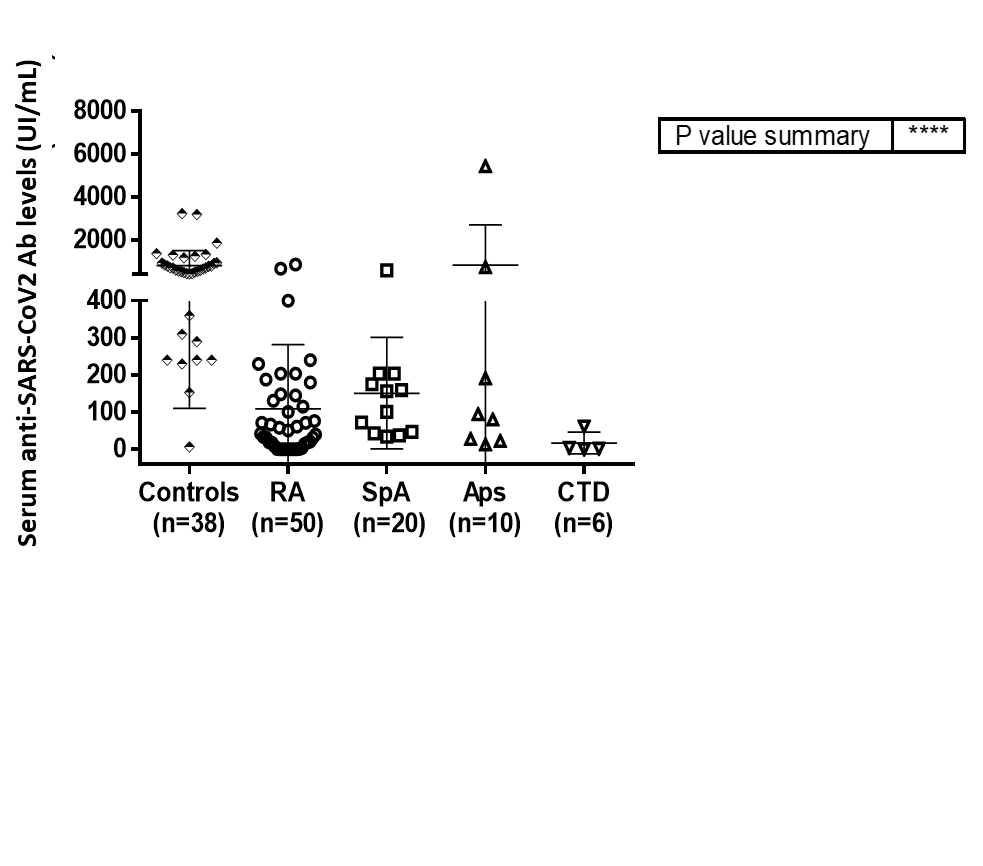
**

**Figure S3**. Proportion of patients with SARS-CoV-2 infections in each therapy group. This graph shows the proportion of patients who developed SARS-CoV-2 infection and its severity after the second dose of vaccine according with each treatment.

**Proportion of patients (%)**


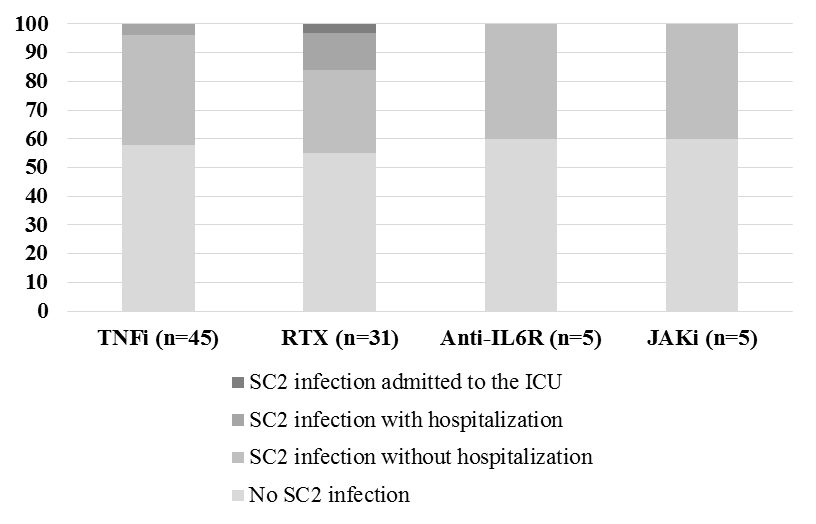


**58%**

**55%**

**60%**

**60%**

**38%**

**29%**

**40%**

**40%**

**4%**

**13%**

**3%**
